# Ultrathin potassiophilic carbon skin design achieving ultra-stable potassium metal anodes†

**DOI:** 10.1039/d5sc04233j

**Published:** 2025-07-23

**Authors:** Zhibin Li, Zheng Hu, Miaoran Deng, Liang Ma, Jinliang Li, Wenjie Mai

**Affiliations:** a Siyuan Laboratory, Guangdong Provincial Engineering Technology Research Center of Vacuum Coating Technologies and New Energy Materials, Guangdong Provincial Key Laboratory of Nanophotonic Manipulation, Department of Physics, College of Physics & Optoelectronic Engineering, Jinan University Guangzhou 510632 China wenjiemai@email.jnu.edu.cn lijinliang@email.jnu.edu.cn; b School of Chemistry, Guangzhou Key Laboratory of Materials for Energy Conversion and Storage, South China Normal University Guangzhou 510006 China maliang2415@m.scnu.edu.cn

## Abstract

Due to the complex fabrication process and poor reversibility of potassium (K) metal, developing high-performance host materials for K metal anodes remains a significant challenge. In this work, an ultrathin and dense N-doped carbon layer was uniformly loaded onto carbon fibers (N-CF) as a host for K metal anodes. This design effectively regulates the intrinsic adsorption behavior of metallic K, mitigating the effects of local uneven electric fields in the electrolyte and enabling stable cycling performance at high current densities. We found that the N functional groups synergistically constructed a robust potassiophilic surface, facilitating spontaneous and rapid integration with molten K. This process effectively suppresses dendrite growth and ensures stable cycling of the K metal anode, even at ultra-high current densities. Thus, the symmetric cell with the N-CF host exhibited remarkable cycling stability, maintaining stable cycling performance over 4300 h at 0.5 mA cm^−2^/0.5 mA h cm^−2^. Furthermore, the anode demonstrated a low polarization voltage and exceptional stability even at 9 mA cm^−2^, underscoring its superior dendrite inhibition capability. Ultimately, the outstanding stability of the N-CF@K metal anode enabled impressive performance in full-cell testing with a Prussian blue cathode. After 300 cycles, the full cell retained a high specific capacity of 91 mA h g^−1^ and a capacity retention of 91.8% at 500 mA g^−1^. We believe that our work offers a novel chance to design an advanced host for achieving stable K metal anode performance at ultra-high current densities.

## Abstract 2

A potassiophilic N-doped carbon skin regulated potassium adsorption and mitigated uneven electric fields, resulting in suppressed dendrite growth and side reactions for K metal batteries.

## Graphical abstract

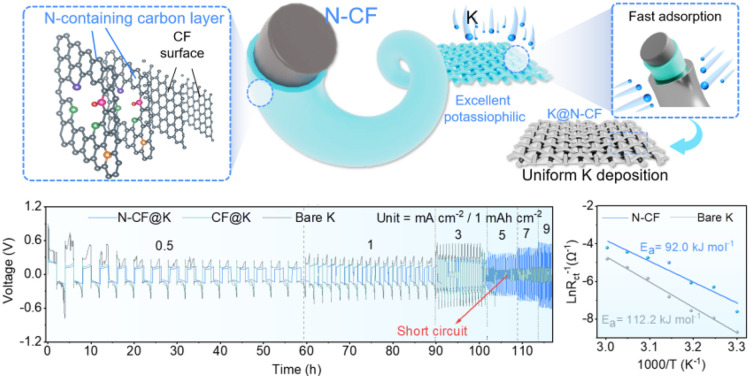

## 1. Introduction

Potassium (K)-ion batteries (KIBs) have garnered significant attention in recent years due to their electrochemical properties being similar to those of lithium-ion batteries, abundant natural reserves and a similar standard redox potential (−2.93 V *vs.* SHE) to lithium.^1^ These compelling advantages establish KIBs as highly promising candidates for next-generation large-scale energy storage applications. Among various anode materials, K metal anodes stand out due to their unparalleled theoretical specific capacity of 687 mA h g^−1^ and the lowest potential plateau.^2^ However, the deposition behavior of K metal is highly sensitive to electric fields. Local uneven electric field distributions in the electrolyte often lead to non-uniform K deposition, resulting in dendrite formation and dead K, which severely hinders the practical application of KIBs.^3^ Addressing the issue of non-uniform deposition remains one of the most pressing challenges in the K metal anode field.

Previous studies have focused on electrolyte regulation to improve the uniformity of K deposition by optimizing the local environment at the electrode–electrolyte interface.^4–8^ However, electrolyte-based approaches still face limitations, such as interface degradation over extended cycling. Consequently, constructing 3D potassiophilic hosts has emerged as an effective strategy to address these challenges.^9–12^ Such hosts facilitate stronger adhesion between K metal and the host material, effectively reducing local current density, and ensuring uniform deposition and stripping. Host materials commonly include metals, carbon, metal–organic frameworks (MOFs), *etc.* Zhang *et al.* developed a Bi nanoparticle modified host for K metal anodes, which maintained a stable voltage hysteresis loop over 550 h with a low overpotential at 0.5 mA cm^−2^/0.5 mA h cm^−2^.^13^ Chen *et al.* designed a carbon nanotube/MOF composite as a host to accelerate the transportation kinetics, achieving a stable performance of 3200 h under 0.35 mA cm^−2^/0.35 mA h cm^−2^ conditions.^14^ However, while metal hosts offer high conductivity, their susceptibility to HF corrosion in fluoride-based electrolytes presents significant drawbacks.^15^ MOFs face challenges related to complex synthesis and insufficient mechanical strength. In contrast, carbon-based materials have emerged as ideal host candidates due to their stability, structural diversity, and cost-effectiveness.^16–19^

Carbon-based hosts can be further functionalized through chemical or physical modifications, such as heteroatom doping or the attachment of potassiophilic materials.^20,21^ For example, Xu *et al.*^22^ deposited SnO_2_ coatings onto carbon nanofibers *via* vapor deposition, achieving enhanced potassiophilicity and structural stability, thereby extending the lifespan of K metal anodes. Similarly, Tu *et al.*^23^ synthesized Ag nanoparticle-loaded carbon cloth (Ag-CC) hosts, effectively regulating K deposition. However, achieving strong and uniform bonding between the host and K metal anode, especially under extreme conditions such as high current densities, still remains critical and challenging. In contrast, heteroatom doping into a carbon host offers a more robust and stable approach. For instance, Mai *et al.*^18^ designed N, H-functionalized carbon cloth, which rapidly and spontaneously adsorbed molten K, forming a stable interface. These findings confirmed that the creation of carbon defects significantly improves potassiophilicity. Despite these advancements, current strategies for the carbon host primarily demonstrate stability at relatively low current densities. Local non-uniformities still persist at high current densities, leading to accelerated dendrite growth and severe interface degradation. Enhancing host stability under high current conditions remains a critical challenge for the practical application of K metal anodes.

**Fig. 1:**
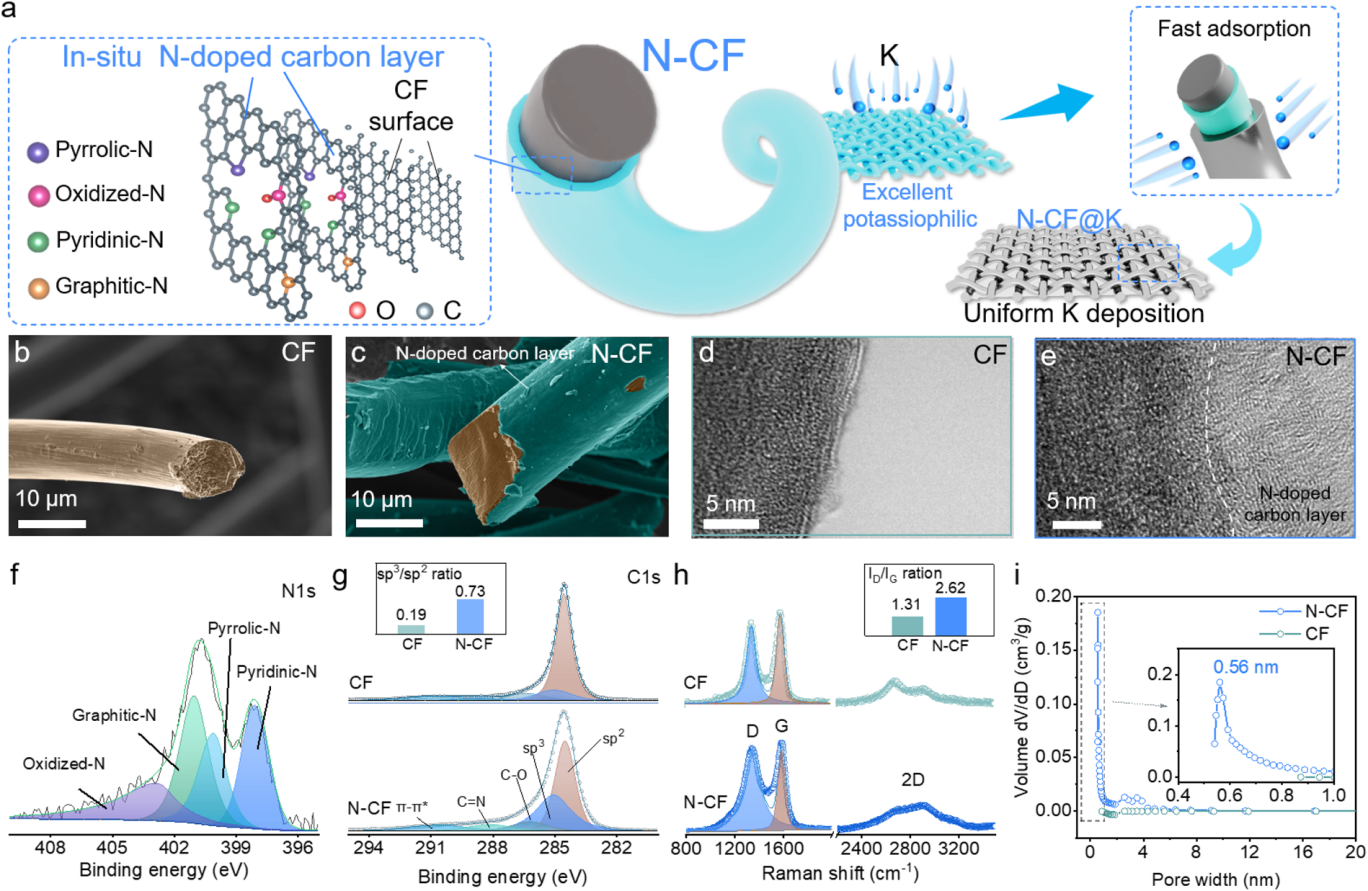
Schematic and characterization of the potassiophilic N-CF mechanism. (a) Schematic of the K deposition on the N-CF host; (b) SEM images of CF and (c) N-CF; TEM images of (d) CF and (e) N-CF interfaces; (f) XPS N 1s spectrum of N-CF; (g) XPS C 1s spectra of CF and N-CF; (h) Raman spectra and (i) BJH pore size distribution of CF and N-CF.

In this study, we engineered a commercial carbon cloth host by depositing an ultrathin and dense potassiophilic N-doped carbon skin onto carbon fibers (N-CF). This modification of the carbon host effectively regulated the intrinsic adsorption behavior of K metal, reducing the impact of local electric field heterogeneities in the electrolyte and enabling stable cycling even at high current densities. Specifically, N-rich polyaniline-derived carbon skin was attached to the carbon fibers, followed by subsequent high-temperature treatment. This process distorted the carbon skeleton, creating abundant defects while retaining N-containing functional groups. These defects and functional groups synergistically constructed a robust potassiophilic surface, which facilitates spontaneous and rapid integration with a K metal anode. As a result, our N-CF host can effectively suppress K dendrite growth and improve the stability of the K metal anode. Theoretical and experimental analyses revealed that the abundant N-containing functional groups and defects in carbon skin significantly enhanced the binding energy between K and N-CF and promoted the desolvation of K ions, thereby inducing uniform K deposition (Fig. 1a). Consequently, the symmetric cell exhibited remarkable cycling stability, maintaining a stable electrochemical performance over 4300 h at 0.5 mA cm^−2^/0.5 mA h cm^−2^ and exceeding 2000 h at 1 mA cm^−2^/0.5 mA h cm^−2^. Even at a high current density of 9 mA cm^−2^, the N-CF host also demonstrated a low polarization voltage and excellent dendrite suppression capability. The used N-CF@K was coupled with a Prussian blue analogue (PBA) as the full cell and it was found that our PBA delivered a high specific capacity of 91 mA h g^−1^ with a capacity retention of 91.8% after 300 cycles at 500 mA g^−1^. These outstanding results highlight the significant potential of N-CF hosts for practical K metal batteries.

## 2. Results and discussion

Considering the unique physicochemical properties of carbon materials in battery systems, commercial carbon fiber cloth (CF) was selected as the precursor material. After a hydrophilic treatment,^24^ the modified carbon fiber cloth (M-CF) exhibited excellent wettability (Fig. S1†) with aqueous solutions. Immersing M-CF in a polyaniline precursor solution ensured sufficient integration of the precursor material (Fig. S2†), and subsequent high-temperature annealing yielded the nitrogen-doped carbon fiber cloth (N-CF). To elucidate the structural features of N-CF, we performed scanning electron microscopy (SEM) of CF and N-CF, as shown in Fig. 1b, c and S3a, b.† Pristine CF exhibits a diameter of ∼9 μm, with carbon being the primary surface component. High-resolution transmission electron microscopy (TEM) images reveal an ordered arrangement of carbon atoms on the pristine CF surface (Fig. 1d) while N-CF presents a similar structure to CF except for the rough interface (Fig. 1e). From the energy-dispersive X-ray spectroscopy (EDS) element mapping, dense N element can be observed, demonstrating that the N-CF surface is covered by a dense N-doped carbon skin (Fig. S3c and d†). TEM analysis further confirms that this carbon skin is ∼80 nm thick (Fig. S3f†), exhibiting a clear boundary with the underlying carbon fiber surface. More disordered carbon skin can be observed, indicating that the N-containing carbon skin introduces numerous pores and defects.^25,26^ Fig. S4† presents the Fourier transform infrared (FTIR) spectra of CF, PANI and N-CF. Compared with CF, typical C–N and C=N bond vibration can be observed, further verifying the introduction of N-containing carbon skin.

We further employed X-ray photoelectron spectroscopy (XPS) to probe the elemental composition and chemical states on the N-CF surface. Fig. S5† presents the survey spectrum of PANI, CF and N-CF. C, N, and O elements can be detected, with a notable increase in nitrogen content (∼4 at%) compared to pristine CF, confirming successful nitrogen doping in the interface. From the high-resolution N 1s spectrum (Fig. 1f), four peaks located at ∼398.1 eV, ∼400.1 eV, ∼401.0 eV, and ∼402.8 eV can be deconvoluted, corresponding to pyridinic-N, pyrrolic-N, graphitic-N, and oxidized-N species, respectively.^27,28^ According to the calculation, pyridinic-N presents the highest content of ∼27 at%. Fig. 1g presents the high-resolution C 1s spectra of CF and N-CF. Six sub-peaks located at ∼284.5 eV (C–C, sp^2^), ∼285.0 eV (“defect peak”, sp^3^), ∼286.2 eV (C–O), ∼288.1 eV (C=N), ∼288.6 eV, and ∼290.7 eV (π–π* satellite peak) were further deconvoluted.^29–31^ Notably, the sp^3^/sp^2^ ratio of carbon increased significantly from 0.19 in CF to 0.73 in N-CF, suggesting the presence of a higher density of defect structures on the N-CF surface.^32,33^ However, no phosphorus signals were detected in the XPS survey spectra of N-CF, indicating that phosphorus primarily acted as a structural promoter during high-temperature annealing, facilitating the formation of active N species.^24,34^Fig. 1h presents the Raman spectra of CF and N-CF. Two typical D and G bands can be observed.^35^ We compared the intensity ratio of the D and G peaks (*I*_D_/*I*_G_), and found that the value of *I*_D_/*I*_G_ increased from 1.31 (CF) to 2.62 (N-CF). Additionally, a distinct 2D peak structure appeared near ∼2700 cm^−1^ in N-CF, attributed to the ultra-thin characteristics of the N-doped carbon skin.^36^ Fig. S6† presents the N_2_ adsorption–desorption isotherms. According to the calculation by the Brunauer–Emmett–Teller method, the specific surface areas of CF and N-CF are 1.6 and 60.0 m^2^ g^−1^, respectively. We also show the fitting result by the Barrett–Joyner–Halenda method to obtain the pore diameter distribution, as shown in Fig. 1i. A pore diameter at ∼0.56 nm in N-CF can be observed. These micropore diameters suggest a significant reorganization of the interfacial pore structure in N-CF, induced by the polyaniline precursor, which incorporated N-containing functional groups facilitated by phytic acid. These comprehensive structural and chemical characterization studies provide crucial insights into the defect-rich nitrogen-doped carbon skin on N-CF, laying the foundation for its excellent performance as a host material for K metal anodes.^24,34,37^

**Fig. 2:**
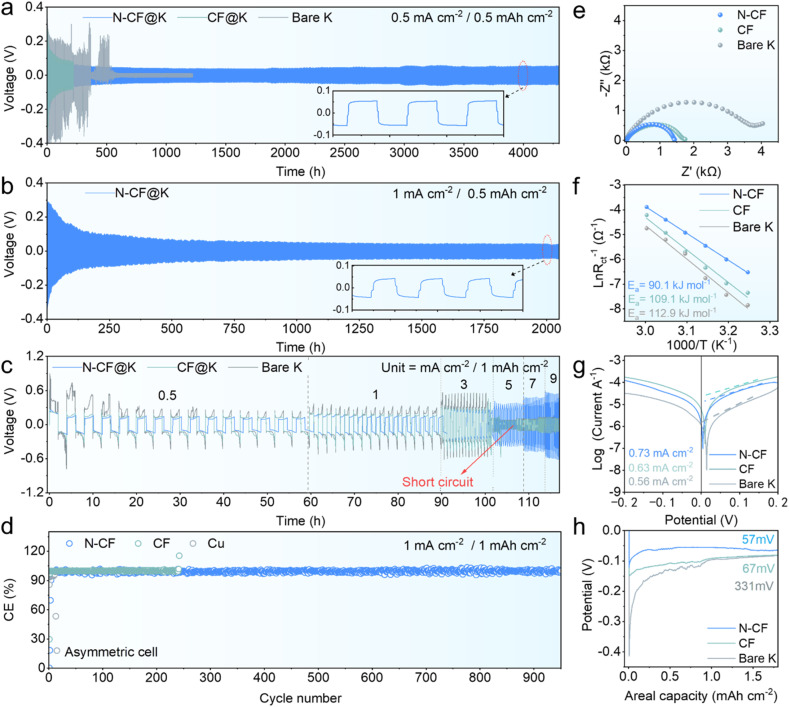
Electrochemical performance. Galvanostatic cycling performance of N-CF, CF and bare K in symmetric cells at (a) 0.5 mA cm^−2^/0.5 mA h cm^−2^; (b) Galvanostatic cycling performance of N-CF at 0.5 mA cm^−2^/1 mA h cm^−2^; (c) cycling performance of N-CF, bare K and N-CF in symmetric cells at different current densities with an areal capacity of 0.5 mA h cm^−2^; (d) CE curves for asymmetric cells N-CF‖K, CF‖K and Cu‖K at 1 mA cm^−2^/1 mA h cm^−2^; (e) Nyquist plot of the symmetric cell before cycling; (f) interface desolvation activation energy fitted using the Arrhenius law of N-CF, CF and bare K; (g) Tafel curve; (h) nucleation overpotential of bare K, CF, and N-CF.

Through N-containing carbon skin modification of CF, the potassiophilicity of the resulting N-CF was significantly enhanced. When molten K was brought into contact with pristine CF for 240 s, no adsorption was observed (Fig. S7a†). In contrast, N-CF achieved uniform and rapid adsorption of molten K within just 0.2 s (Fig. S7b†), demonstrating its strong potassiophilicity. This rapid adsorption of molten K metal offers advantages over electrodeposition, including a more uniform K distribution, less aggressive to preserve structural integrity. While electrodeposition provides precise localization and controlled morphology, adsorbing molten K metal is more effective in achieving uniform K distribution while preserving the structural and conductive features of the N-CF host. Moreover, the adsorption method is operationally simpler and more amenable to large-scale application. Molten K was subsequently adsorbed onto N-CF (denoted as N-CF@K) for electrochemical testing. Since pristine CF could not effectively adsorb molten K, mechanical rolling was employed to combine CF with K (denoted as CF@K). Fig. S8† displays front and side views of bare K, CF@K, and N-CF@K electrodes. Symmetric cells were assembled using bare K‖bare K, CF@K‖CF@K, and N-CF@K‖N-CF@K configurations to evaluate K deposition behavior, as shown in Fig. 2a. It is found that the bare K‖bare K cell only exhibited significantly large voltage polarization at 0.5 mA cm^−2^/0.5 mA h cm^−2^ during the initial cycles, indicating the formation of extensive dead K and severe interface degradation. When CF was used as a host, the CF@K‖CF@K cell demonstrated improved cycling stability compared to bare K‖bare K, with a lifespan of 223 h under the same conditions (Fig. S9a†). However, the N-CF host enabled stable cycling for up to 4300 h at 0.5 mA cm^−2^/0.5 mA h cm^−2^, marking a significant improvement in cycle life. To further verify the stability at large current density, we also evaluate the cycling performance of the N-CF@K‖N-CF@K cell at 1 mA cm^−2^/0.5 mA h cm^−2^. The cell also enabled stable cycling for up to 2050 h (Fig. 2b). We also provide the voltage profiles of the bare K‖bare K, CF@K‖CF@K, and N-CF@K‖N-CF@K cells at 0.5, 1, 3, 5, 7, and 9 mA cm^−2^ with a capacity of 1 mA h cm^−2^ (Fig. 2c). It is found that the bare K‖bare K cell exhibited severe voltage polarization during the initial cycles, rendering further testing impractical. The CF@K‖CF@K cell short-circuited at 3 mA cm^−2^, indicating poor stability at high current densities. In contrast, the N-CF@K‖N-CF@K cell maintained stable cycling even at an ultra-high current density of 9 mA cm^−2^. Fig. S9b† presents the enlarged voltage profiles of N-CF at current densities from 3–9 mA cm^−2^. The stability of N-CF@K metal anode cycling at 9 mA cm^−2^ is better than that of all previously reported K metal cathodes (Fig. S10a†). This result indicates that the bottleneck that K metal anodes cannot tolerate high current is expected to be solved. In addition, a comparison with previously published studies (Fig. S10b†) highlights that the N-CF host delivers superior cycling performance.

To assess the reversibility of K plating/stripping, the cycling performance of K‖Cu, K‖CF, and K‖N-CF asymmetric cells was tested, as shown in Fig. 2d. The K‖Cu cell only exhibited a sharp drop in coulombic efficiency (CE) to 18.1% after just 15 cycles at 1 mA cm^−2^/1 mA h cm^−2^. Even at 0.5 mA cm^−2^/1 mA h cm^−2^, the CE of the K‖Cu cell dropped significantly after 30 cycles (Fig. S11a†), indicating poor reversibility on the Cu host. The corresponding GCD curves (Fig. S11b†) show significant interface degradation after 100 h. In contrast, the K‖CF cell exhibited an improved cycle life of 241 cycles at 1 mA cm^−2^/1 mA h cm^−2^, but its CE rapidly declined thereafter (Fig. S12a†). After 490 h, pronounced fluctuations in the GCD curve indicated extensive dead K formation, further deteriorating reversibility.^38^ Notably, the K‖N-CF cell displayed significantly enhanced reversibility, maintaining an exceptional average CE of 99.1% even after 970 cycles. The corresponding GCD curves (Fig. S12c and d†) remained smooth and nearly overlapping, further confirming the excellent reversibility of K plating/stripping on the N-CF host.

Electrochemical impedance spectroscopy (EIS) measurements in Fig. 2e revealed electron transfer resistances of 3501, 1505, and 1228 Ω for bare K, CF@K, and N-CF@K, respectively according to the circuit diagram in Fig. S13,† highlighting the role of N-CF in effectively reducing interfacial resistance and polarization. To further evaluate dynamic characteristics, EIS of bare K, CF and N-CF at varying temperatures was performed (Fig. S14†). According to the Arrhenius equation, we calculated the K desolvation activation energies of 112.9 kJ mol^−1^, 109.1 kJ mol^−1^ and 90.1 kJ mol^−1^ for bare K, CF and N-CF, respectively, confirming that the N-CF host facilitates rapid K ion desolvation and reduces local ion accumulation (Fig. 2f). Tafel plots in Fig. 2g indicate exchange current densities of 0.56, 0.63, and 0.73 mA cm^−2^ for bare K, CF, and N-CF, respectively. The higher exchange current density observed for N-CF suggests more favorable reaction kinetics, mitigating the slow deposition issue commonly observed with K metal anodes. However, the exchange current density of N-CF remains lower than that of CF hosts. The poor potassiophilicity of CF results in uneven K distribution. Fig. 2h shows the voltage profiles during initial K plating, revealing nucleation overpotentials of 331, 67, and 57 mV for bare K, CF, and N-CF, respectively. The lower overpotential observed for N-CF indicates a more favorable K deposition process, further underscoring its potential as an optimal host for stable K metal batteries.

**Fig. 3:**
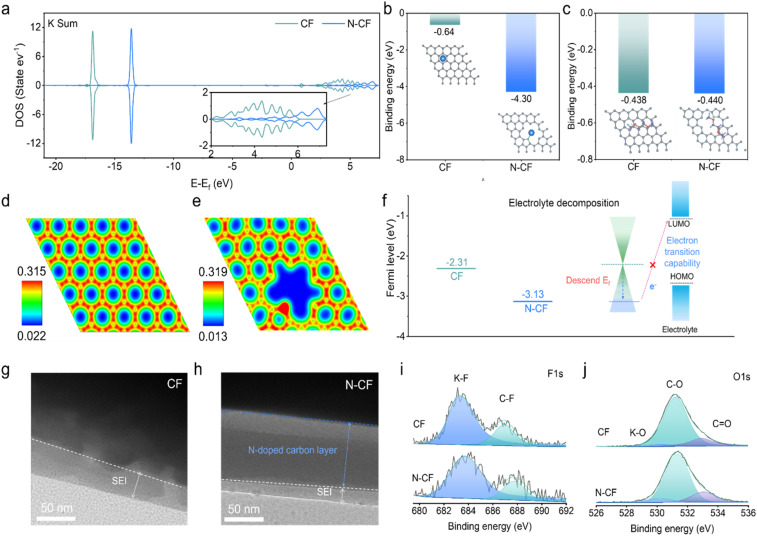
DFT calculations and the corresponding interface characterization. (a) DOS results of the K orbitals for CF and N-CF; surface binding energies of CF and N-CF to (b) K and (c) DME molecules. Charge density of (d) CF and (e) N-CF on the surface. (f) Plots of the surface Fermi levels of the host material relative to the electrolyte; TEM images of the SEI layers of (g) CF and (h) N-CF after 5 cycles; (i) F 1s and (j) O 1s XPS spectra of SEI layers of CF and N-CF after 5 cycles.

To further understand the underlying mechanism behind the superior electrochemical performance of N-CF, density functional theory (DFT) simulations were performed. Given that pyridinic-N dominates the N doping in N-CF, the N-CF model primarily considered the pyridinic N doping into carbon skin. The corresponding configurations of CF and N-CF with a K atom and electrolyte are shown in Fig. S15.† From the total density of states (TDOS) and the projected density of states (PDOS) for N-CF and CF with a K atom (Fig. S16†), a significant shift of N-CF's hybridized orbitals toward higher energy levels compared to those of CF is found. This result indicates more obvious cationic properties of N-CF with a K atom. Further analysis of the PDOS for K orbitals on CF and N-CF (Fig. 3a) shows that the DOS of N-CF broadens in the high-energy region. This broadening facilitates stronger orbital hybridization between K and N, enabling electron transfer from the electron-enriched N sites to the electropositive K atoms. Therefore, the presence of pyridinic-N significantly enhances K adsorption and deposition at the N-CF interface. Fig. 3b compares the binding energy of K atoms on CF and N-CF surfaces. N-CF exhibits a significantly higher binding energy of −4.30 eV compared to −0.64 eV for CF, confirming the enhanced potassiophilicity of the N-CF interface. Additionally, we also calculate the binding energy between dimethoxyethane (DME) solvent molecules and N-CF/CF, as shown in Fig. 3c. The results indicate no substantial difference between N-CF and CF in solvent binding energy, which supports the experimentally observed lower desolvation activation energy for N-CF, facilitating rapid K ion desolvation. The electronic density maps for CF and N-CF surfaces are shown in Fig. 3d and f. The CF surface displays relatively uniform electron density distribution across carbon atoms, whereas N-CF shows a significant increase in electron density in regions of pyridinic-N induced carbon defects. This local electron density increase facilitates an electronic interaction effect, enhancing the electrochemical interface stability. A comparison of the Fermi levels of CF and N-CF (Fig. 3f) reveals values of −2.31 eV and −3.13 eV, respectively. The larger energy difference between N-CF's Fermi level and the LUMO energy level of the DME electrolyte indicates that electron transfer from N-CF to the electrolyte is significantly suppressed. This suppression reduces parasitic side reactions during K plating/stripping and improves the electrochemical stability of the K metal anode. Besides, the comparison of the bond energy change (Δ*E*_b_) for N-CF (0.60 eV) and CF (0.72 eV) showed the more efficient formation of KF *via* S–F bond cleavage in N-CF (Fig. S17†).

To validate the computational results, post-cycling electrode surfaces were characterized using TEM and XPS. From TEM images in Fig. 3g and h, it is found that the thickness of the SEI layer on the CF surface is ∼50 nm, whereas the SEI layer on the N-CF surface is significantly thinner (∼20 nm) and more uniform. Further XPS analysis of SEI composition (Fig. 3i) indicates that both CF and N-CF surfaces are primarily composed of K–F (∼683.7 eV) and C–F (∼687.8 eV) species, as observed in their F 1s spectra.^2^ Their O 1s spectra also reveal peaks corresponding to K–O (∼530.2 eV), C–O (∼531.3 eV), and C=O (∼533.1 eV).^39^ From the XPS results, we find that the N-CF host contributes to the formation of an abundance of K–F, suggesting the robust inorganic-rich SEI layer in N-CF, which typically enhances SEI mechanical strength and stability.^40^ This inorganic composition is likely derived from the KFSI ether-based electrolyte used in the study.

**Fig. 4:**
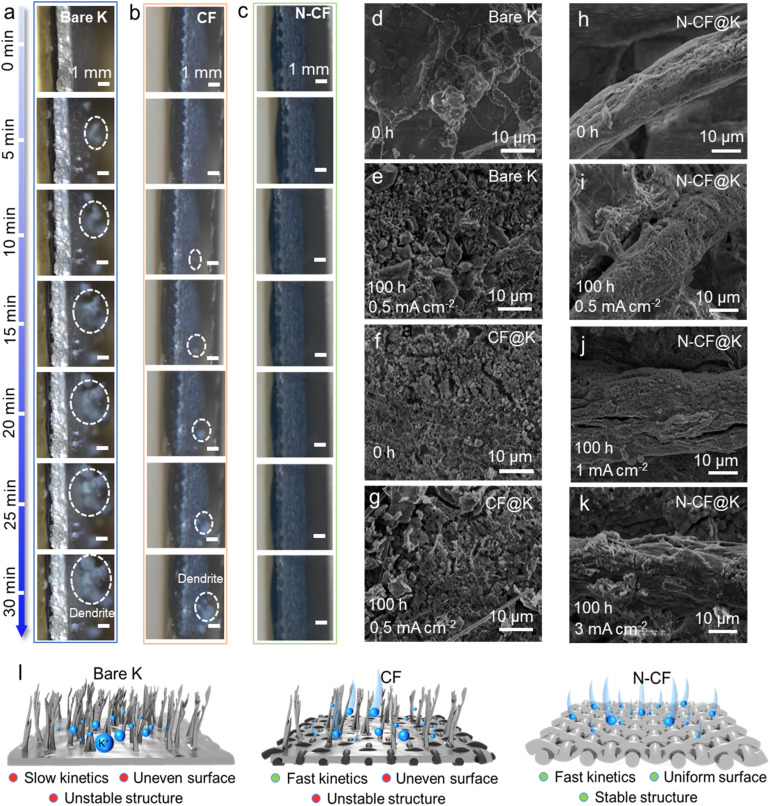
Process and mechanism of the K metal plating–stripping process. *In situ* optical microscope photographs for (a) bare K, (b) CF@K and (c) N-CF@K at a plating areal current density of 0.6 mA cm^−2^. SEM images of (d and e) bare K, (f and g) CF@K and (h–k) N-CF@K before and after cycling; (l) schematic diagram of the deposition kinetics for bare K, CF, and N-CF.

The unique N-containing carbon skin of N-CF endows it with remarkable potassiophilicity, superior interfacial kinetics, and minimized side reactions. These synergistic effects collectively enable more uniform and stable K plating/stripping, ultimately leading to exceptional electrochemical performance. To gain deeper insights into the K plating/stripping behavior on N-CF, we employed *in situ* optical microscopy for characterization. As shown in Fig. 4a–c, *in situ* optical microscopy was used to monitor K deposition on different hosts at 0.6 mA cm^−2^ for 30 min. On bare K, substantial dendrite formation and K accumulation were observed, indicating highly irregular deposition. Similarly, the CF host also exhibited notable dendritic structures. In contrast, the N-CF host displayed neither dendrites nor localized K accumulation, highlighting the ability of N-CF to effectively guide uniform K deposition and suppress dendrite formation. These findings are further corroborated by post-cycling electrode analysis (Fig. S18†). After 100 h of cycling at 0.5 mA cm^−2^/0.5 mA h cm^−2^, significant dead K and dendrite buildup were observed on bare K and CF@K and their corresponding separators. In contrast, nucleation is clearly visible along the lateral surfaces of N-CF after 30 min of plating, confirming effective metal deposition within the host, and the N-CF@K surface remained relatively smooth and uniform, underscoring its superior ability to regulate K deposition during prolonged cycling. SEM imaging further elucidated the host surface deposition behavior, as shown in Fig. 4d–g. Before deposition, bare K and CF@K displayed irregular K distribution, while N-CF showed a uniform layer of adsorbed K across the carbon fiber surface. This uniform adsorption persisted throughout the deposition process, enabling consistent K deposition even after 100 h of cycling at current densities of 0.5, 1, and 3 mA cm^−2^ (Fig. 4h–k). In contrast, significant surface cracking and fragmentation were evident on bare K and CF@K after 100 h of cycling at 0.5 mA cm^−2^. To complement these experimental observations, finite element analyses (FEA) were performed to model K deposition dynamics on different hosts, as shown in Fig. S19.† The simulations incorporated the N doping and carbon defect structures of N-CF, as well as the relatively K-phobic surface of CF. By assigning uniform and non-uniform initial exchange current densities to N-CF and CF, respectively, the simulations revealed that the non-uniform current density distribution on CF led to more irregular K-ion concentration distribution over time. Conversely, the uniform and stable electron flux on N-CF directed the current density evenly across the host surface, facilitating controlled K deposition and effectively suppressing dendrite growth. These findings align closely with the results from *in situ* optical microscopy and SEM characterization.

Based on these observations, we propose a schematic illustration of the deposition kinetics for bare K, CF, and the N-CF host. On bare K, the absence of an underlying structural template leads to uneven K deposition, rapid dendrite growth, and an unstable interface. During stripping, this unstable interface promotes dead K formation, resulting in slow kinetics and structural instability. When a CF host is introduced, its electronic conductivity is significantly enhanced; however, it fails to form a stable and uniform interface, still leading to structural instability. In contrast, the N-CF host combines fast kinetics with strong potassiophilicity, enabling uniform K deposition, stable interface formation, and prolonged structural stability. This ultimately ensures superior cycling stability in K metal batteries.

**Fig. 5:**
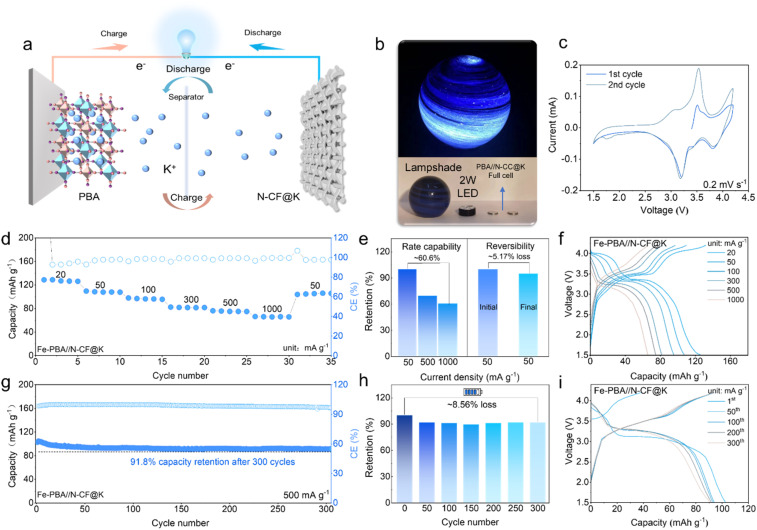
Electrochemical performance of the PBA‖N-CF@K full cell. (a) Schematic diagram; (b) application demonstration; (c) CV curve; (d) rate performance and (e) rate capacity retention statistics; (f) corresponding GCD curve; (g) long-term cycle performance; (h) capacity retention statistics; (i) corresponding GCD curves.

To demonstrate the practical applicability of our K metal battery system, we paired the N-CF@K anode with a PBA cathode to construct an N-CF@K‖PBA full cell, as shown in Fig. 5a. Impressively, the N-CF@K‖PBA cell successfully powered a 2 W LED light (Fig. 5b), highlighting its potential for real-world energy storage applications. The cyclic voltammetry (CV) curve of the N-CF@K‖PBA cell (Fig. 5c) reveals two distinct redox peaks, corresponding to the reversible redox reactions of low-spin Fe^LS^–C and a high-spin Fe^HS^–C voltage plateau. To evaluate the electrochemical performance of the N-CF@K‖PBA cell, rate performance was evaluated over the voltage range of 1.5–4.2 V at varying current densities (Fig. 5d). The cell delivered a specific capacity of 128 mA h g^−1^ at 20 mA g^−1^. Even at a high current density of 1 A g^−1^, it maintained a capacity of 66 mA h g^−1^, corresponding to a retention rate of 66.6% (Fig. 5e). Fig. 5f shows the corresponding GCD curves at varying current densities. After a series of rate capability tests, the capacity recovered to 50 mA g^−1^ with only a 5% capacity loss, while the charge–discharge profiles remained stable with a consistent high-voltage plateau. Long-term cycling performance was further evaluated at 500 mA g^−1^ (Fig. 5g). Remarkably, the N-CF@K‖PBA cell retained a specific capacity of 91 mA h g^−1^ after 300 cycles, with a capacity degradation of only 8.56% (Fig. 5h). The overlapping charge–discharge profiles throughout cycling (Fig. 5i) further confirm the outstanding reversibility and cycling stability of the N-CF@K‖PBA cell.

## 3. Conclusion

In this work, we design a N-containing carbon skin on CF to serve as a host for K metal anodes. We find that this configuration effectively manages the adsorption behavior of metallic K, reducing the impact of uneven electric fields in the electrolyte and enhancing stability at high current densities. The N functional groups play a crucial role in constructing a potassiophilic surface, which promotes rapid integration with molten K and suppresses dendrite growth. As a result, the symmetric cell with the N-CF host demonstrated exceptional cycling stability. Remarkably, the N-CF host also exhibited low polarization voltage and stability even at a high current density of 9 mA cm^−2^, highlighting its superior dendrite inhibition. We believe that this work presents a novel approach for designing advanced hosts for achieving stable K metal anode performance at ultra-high current densities.

## Author contributions

Z. Li performed investigation, methodology, data curation, wrote the original draft. Z. Hu, M. Deng performed investigation. L. Ma, W. Mai and J. Li performed supervision, conceptualization and wrote, review & edited the final manuscript.

## Conflicts of interest

There are no conflicts to declare.

## Supplementary Material

SC-OLF-D5SC04233J-s001

## Data Availability

The data supporting this article have been included as part of the ESI.†
