# Supplementary material for: Ultrathin potassiophilic carbon skin design achieving ultra-stable potassium metal anodes
Source: Chem Sci. 2025 Jul 23;16(34):15537–45. doi: 10.1039/d5sc04233j (PMC12308916; doi:10.1039/d5sc04233j)
Supplement: SC-016-D5SC04233J-s001 [file SC-016-D5SC04233J-s001.pdf]

Supplementary Information (SI) for Chemical Science.
This journal is © The Royal Society of Chemistry 2025

Supporting information

Ultrathin potassiophilic carbon skin design achieving ultra-stable potassium metal anode

Zhibin Li[a], Zheng Hu[a], Miaoran Deng[a], Liang Ma[b], Jinliang Li*[a] , and Wenjie Mai*[a,d]*

[a] Siyuan Laboratory, Guangdong Provincial Engineering Technology Research Center of Vacuum Coating Technologies and New Energy Materials, Guangdong Provincial Key Laboratory of Nanophotonic Manipulation, Department of Physics, College of Physics & Optoelectronic Engineering, Jinan University, Guangzhou 510632, China.

[b] School of Chemistry, Guangzhou Key Laboratory of Materials for Energy Conversion and Storage, South China Normal University, Guangzhou, 510006, China

* Corresponding author: maliang2415@m.scnu.edu.cn (L. Ma), lijinliang@email.jnu.edu.cn (J. Li); wenjiemai@email.jnu.edu.cn (W. Mai)

Methods

1. Experimental Section

1.1 Synthesis of M-CF.

Typically, 0.5 mmol of potassium persulfate and 0.15 mmol KOH was dissolved into 100 mL deionized water to form solution. The CF was immersed into the solution in autoclave and heated to 180 °C at a rate of 10 °C min^{-1} and maintained at 180 °C for 12 h. The M-CF was obtained by washing the production with deionized water and ethanol for several times and dried it at 80 °C for 12 h.

1.2 Synthesis of N-CF.

Typically, polyaniline (PANI) was synthesized as follows: First, 2.4 mL of aniline monomer was dissolved in 10 mL of 50% phytic acid to form Solution A. Separately, 1 mM ammonium persulfate (APS) was dissolved in 50 mL of deionized water to form Solution B. PANI was then obtained by mixing solutions A and B. The M-CF substrate was immersed in the PANI solution for infiltration, followed by carbonization at 900 °C under an Ar atmosphere. The resulting material was dried at 60 °C and designated as N-CF.

1.3 Synthesis of Prussian blue analogue (PBA) cathode materials.

In a typical synthesis procedure, 2 mmol of $FeCl_3$ was dissolved into 160 mL of deionized water to form solution C. 4 mmol of $K_4[Fe(CN)_6]$·3H2O, 0.1 g of ascorbic acid were dissolved into 40 mL of deionized water to form solution D. Under N_2 bubbling and continuous string, solution C was added dropwise into solution D at a rate of 1 mL min^{-1}. The mixture was stirred for 2 h and aged for another 24 h. PBA powder can be obtained by subsequently cooling down, centrifugation, washing, and drying processes.

2. Characterization

The morphologies of the electrode were obtained by scanning electron microscope (SEM, FEI), transmission electron microscopy (TEM, TEOL), and optical microscope (Leica). The structures and surface properties were measured by X-ray diffraction (XRD, D8 Rigaku-9000), Raman spectrometer (Horiba T6400), and X-ray photoelectron spectroscopies (XPS, Thermo Scientific).

For the in-situ visualization observation, we used a portable optical microscope and cooperated with an electrochemical reaction cell to realize the in situ observation of the electrode.

3. Electrochemical measurement

The electrochemical performances of all the samples were tested with coin-type cells (CR2032), which were assembled in an argon-filled (O_2<0.1 ppm, H_2O<0.1 ppm) glove box (Etelus Lab2000) 5 M potassium bis(fluorosulfonyl)imide dissolve into dimethoxyethane (DME) was used as electrolyte. A piece glass fiber (diameter: 18 mm, Whatman GF/C) was used as the separator with 150 µL electrolyte to ensure the separator was fully permeated. The symmetric cell was assembled by the same electrode at both ends of the cell. The half cell of Cu//K, CF//K and N-CF//K was assembled with one piece of K foil (14 mm) as the counter electrode and a work electrode of Cu, CF and N-CF (14 mm). The full cell was assembled with one piece of PBA as the cathode and a N-CF @K as the anode. The PBA cathode was prepared by mixing the active materials(70 wt%), super-P (20wt%), and poly(vinylidene fluoride) binder (10 wt%) in N-methyl-2-pyrrolidinone to form a homogeneous slurry, which was pasted on Al foil and then dried in a vacuum oven at 120 °C for 12 h. The mass loading of the PBA cathode was 1~2 mg cm^{-2}. The GCD curves were tested by the Neware battery test system (Neware BTS-4000). The EIS, Tafel plots and nucleation overpotential of the symmetric cells were recorded by Chenhua electrochemical workstation (CHI660D). The activation energy for de-solvation (E_{ct}) during K deposition process was calculated by Arrhenius equation:

$$\frac{T}{R_{ct}} = Aexp\left(\frac{E_{ct}}{RT}\right)$$

where R, T, A, and R_{ct} are the standard gas constant, temperature, pre-exponential factor, and the fitting resistance of symmetric cells at different temperatures.

4. DFT computational methods

All the calculations were performed in the framework of the density functional theory with the projector augmented plane-wave method, as implemented in the VASP. The generalized gradient approximation proposed by Perdew, Burke, and Ernzerhof screened hybrid density

functional were selected for the exchange-correlation potential. The van der Waals interaction was described by the DFT-D3 approach. The cut-off energy for plane wave was set to 350 eV. The energy criterion was set to 10^{-5} eV in iterative solution of the Kohn-Sham equation. A vacuum layer of 20 Å was added perpendicular to the sheet to avoid artificial interaction between periodic images. The Brillouin zone integration was performed using a 2 x 2 x 1 K-mesh. All the structures were relaxed until the residual forces on the atoms have declined to less than 0.02 eV $Å^{-1}$.

The binding energy (E_b) was calculated via the equation: $E_b = E_{tot} – E_{mater} – E_X$, where E_{tot} was the total energy of the adsorbed compounds, E_{mat} was the energy of CF and N-CF, and E_X was the energy of the K ion and DME. The Fermi energy (E_f) was calculated by the single-point energy of CF and K-CF.

The bond energy change (ΔE_b) of S–F bond cleavage was calculated with the equation: $\Delta E_b = E_{sub\text{-}(FSI2)} - E_{sub\text{-}(FSI1)}$, where $E_{sub\text{-}(FSI2)}$ represents total bond energy in the anion of FSI^- after defluorination on a substrate, and $E_{sub\text{-}(FSI1)}$ represents total bond energy in the anion of FSI^- on a substrate (CF or N-CF).

5. FEA computational methods

The finite element analysis of the ion concentration on the surface of the CF and N-CF anodes during the process of potassium ion deposition were simulated using the multiple models of tertiary current distribution, nernst-planck (t_{cd}) interface and the deformed geometry (d_g) interface. The ultra refinement computational grids were controlled by the physics precisions. To reflect the difference in structure, the local exchange current density of CF anode surface was uneven, while the local exchange current density of N-CF anode surface was even. In order to ensure the consistency of reaction conditions, the potassium deposition time used and total current density by the two anodes were consistent.

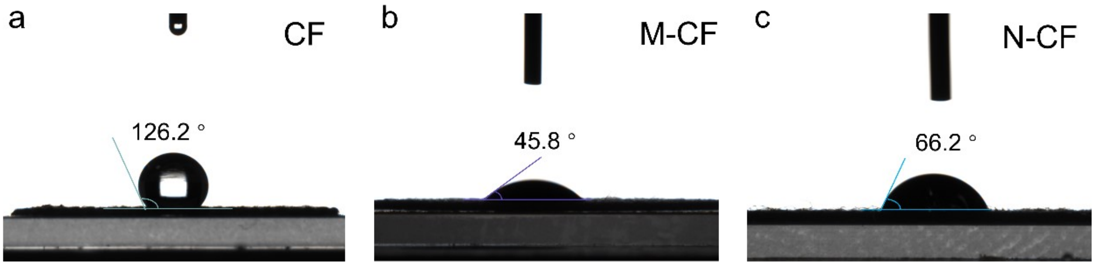

Figure S1 The hydrophilicity tests of (a) CF, (b) M-CF, and (c) N-CF.

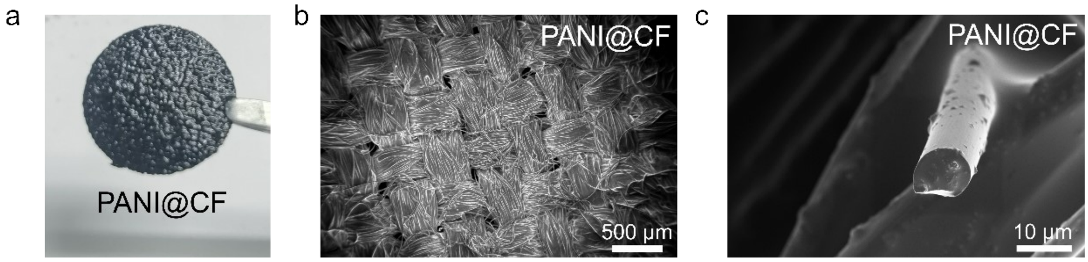

Figure S2 (a) Optical images, (b) SEM and (c) enlarged SEM images of PANI@M-CF.

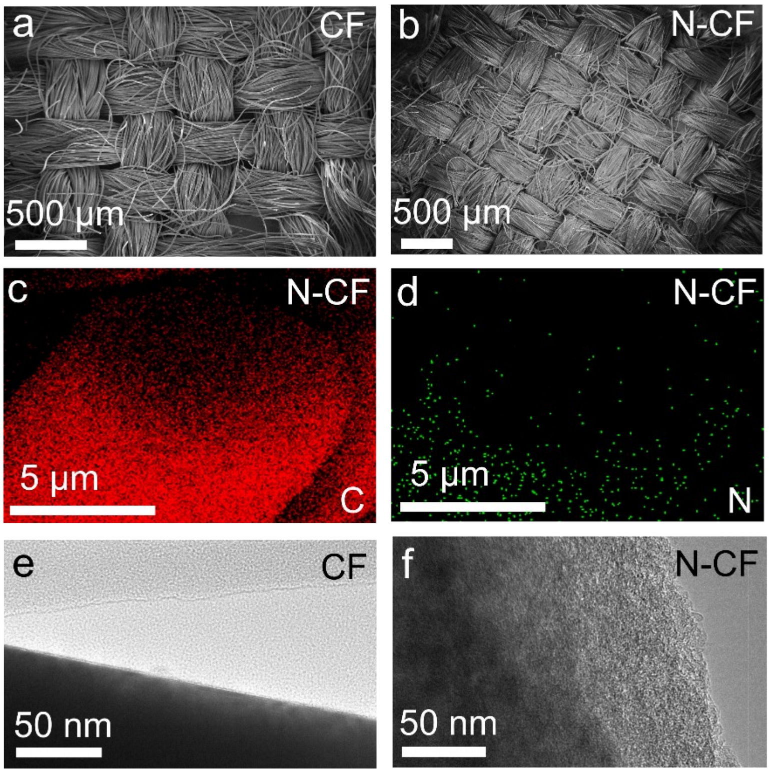

Figure S3 SEM images (a-b) of CF and N-CF;(c-d) EDS spectra of N-CF; TEM images (e) CF and (f) N-CF.

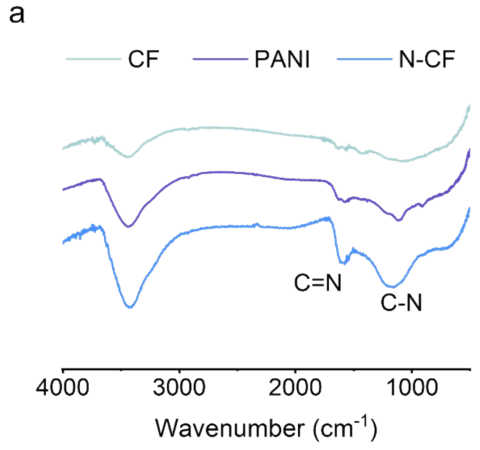

Figure S4 FTIR spectra of CF, PANI, and N-CF.

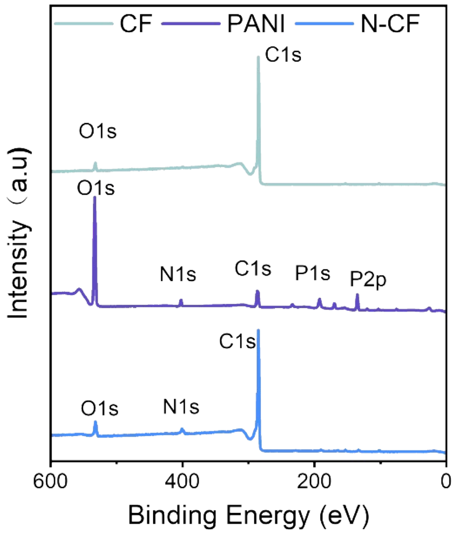

Figure S5 XPS spectrum of PANI, CF and N-CF.

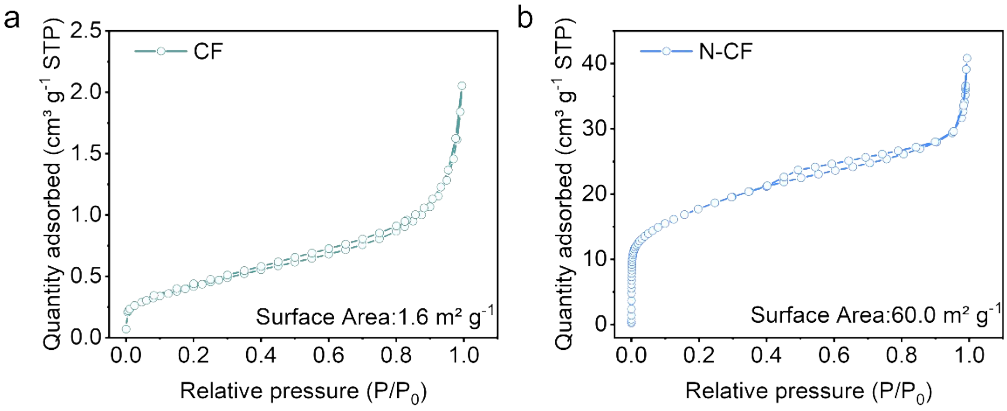

Figure S6 N_2 adsorption-desorption isotherms of (a) CF and (b) N-CF.

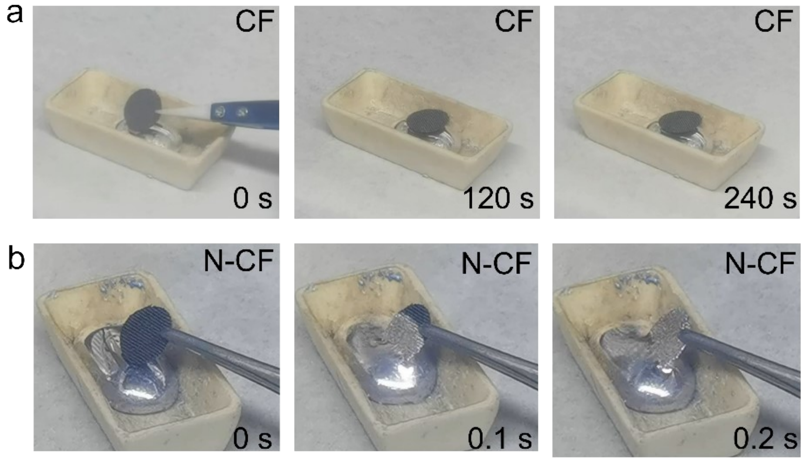

Figure S7 Adsorption process of molten potassium metal on (a) CF and (b) N-CF.

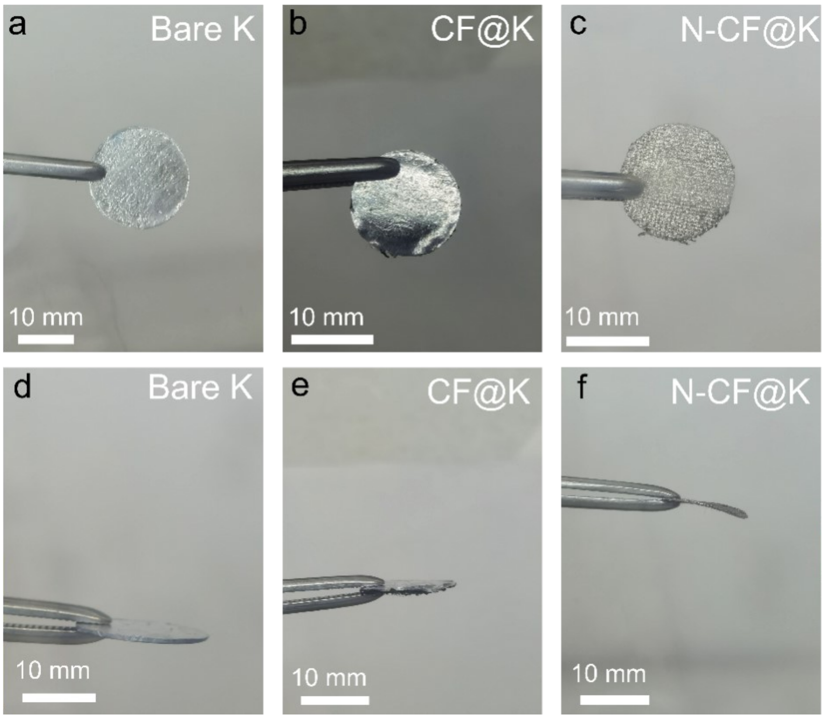

Figure S8 Front and side views of the electrodes: (a, d) Bare K, (b, e) CF@K, and (c, f) N-CF@K.

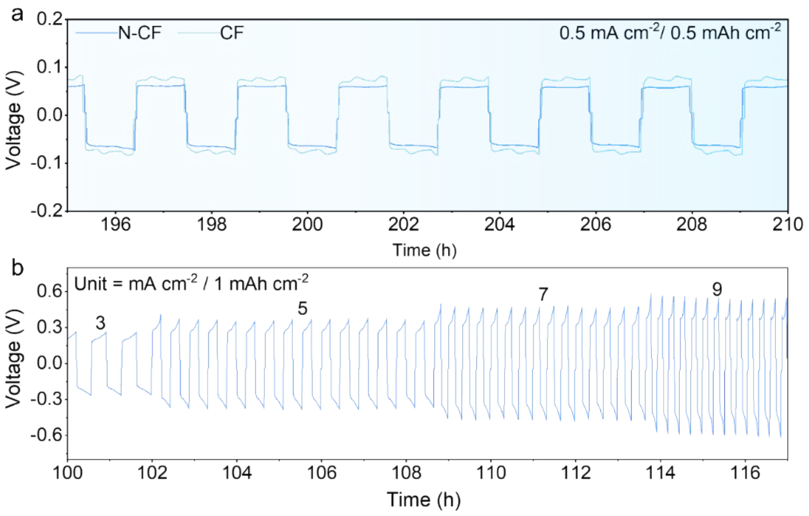

Figure S9 Enlarger view of galvanostatic cycling performance in Figure 2a and (b) Figure 2c.

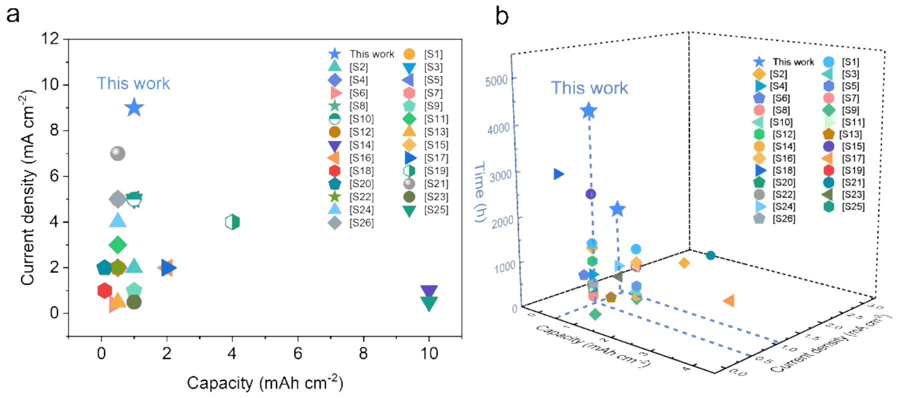

Figure S10 Comparison of (a) rate performance and (b) cycling stability between N-CF and previously reported host materials.

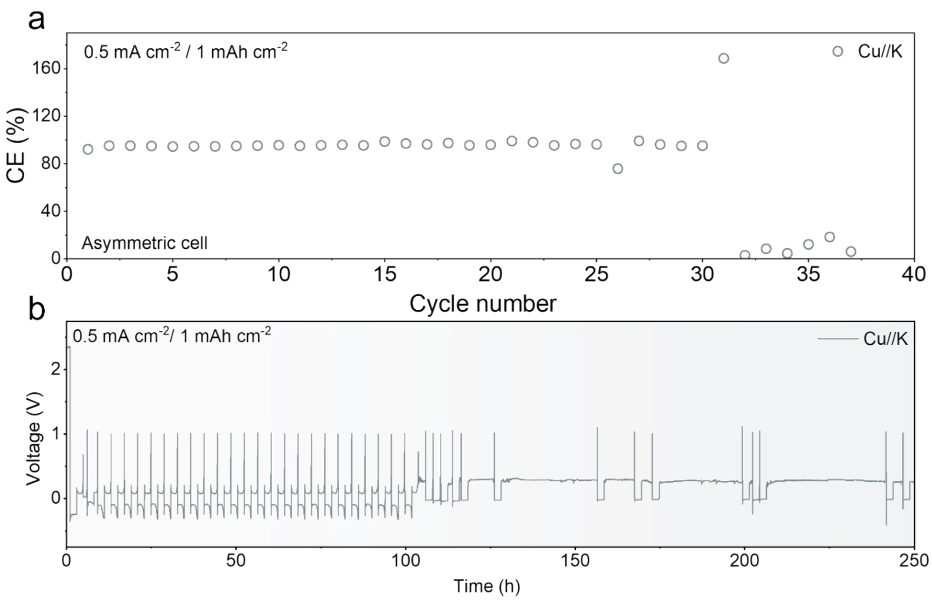

Figure S11 (a) CE of asymmetric cells for Cu at 0.5 mA cm^{-2}/ 0.5 mAh cm^{-2} and (b) corresponding GCD curves.

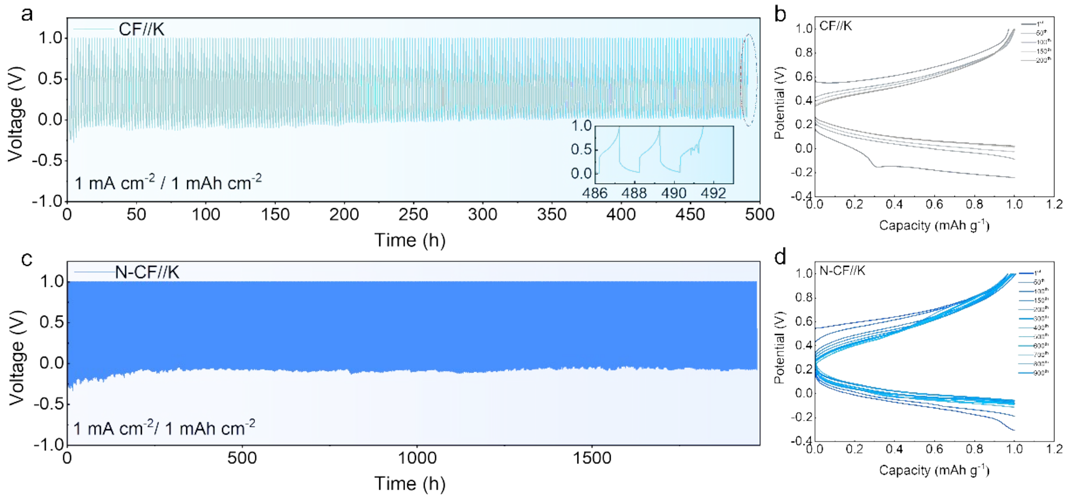

Figure S12 (a) GCD curves of the asymmetric cells for K||CF and (c) K||N-CF at a current density of 1 mA cm^{-2} / 1 mAh cm^{-2};(b) Capacity-voltage curves of K||CF and (d) K||N-CF at different cycle numbers.

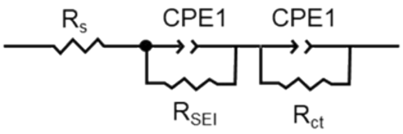

Figure S13 Circuit diagram for EIS fitting the electron transfer resistances in Figure 2e.

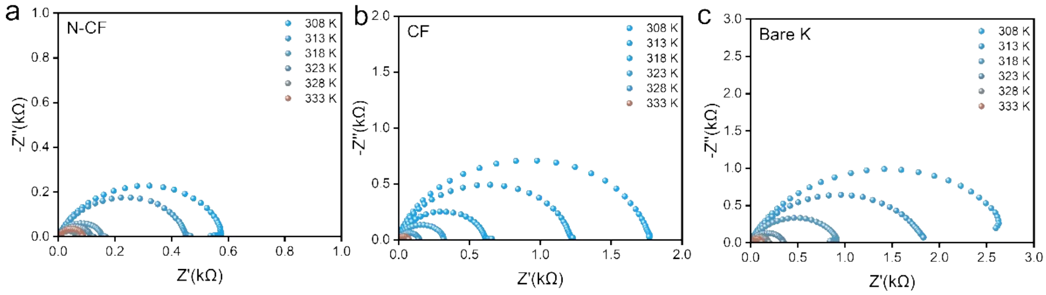

Figure S14 Nyquist plots of symmetric cells at different temperatures: (a) N-CF@K, (b) CF@K, and (c) Bare K.

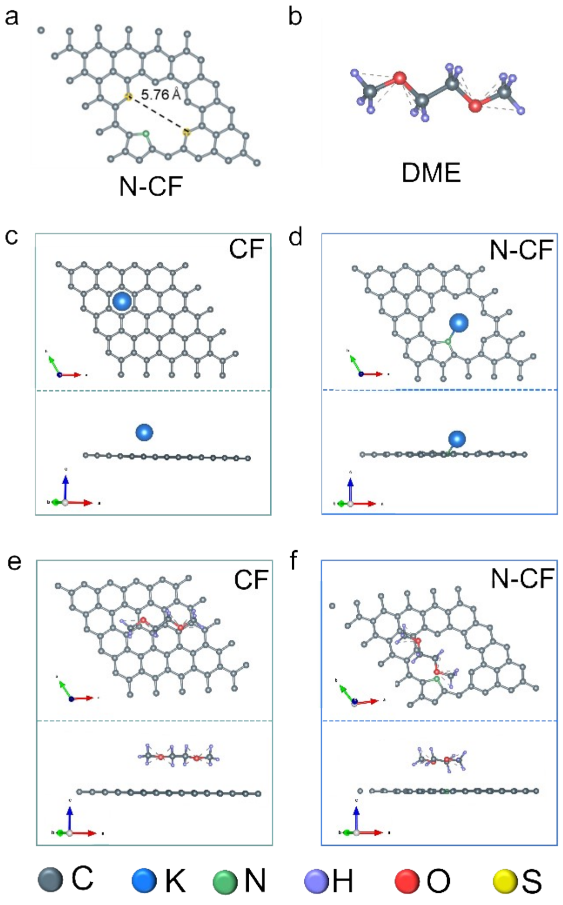

Figure S15 Structural models in DFT calculations: (a) Atomic structure of the N-CF surface, (b) DME molecule, (c) Potassium adsorption on CF, (d) Potassium adsorption on N-CF, (e) DME adsorption on CF, and (f) DME adsorption on N-CF.

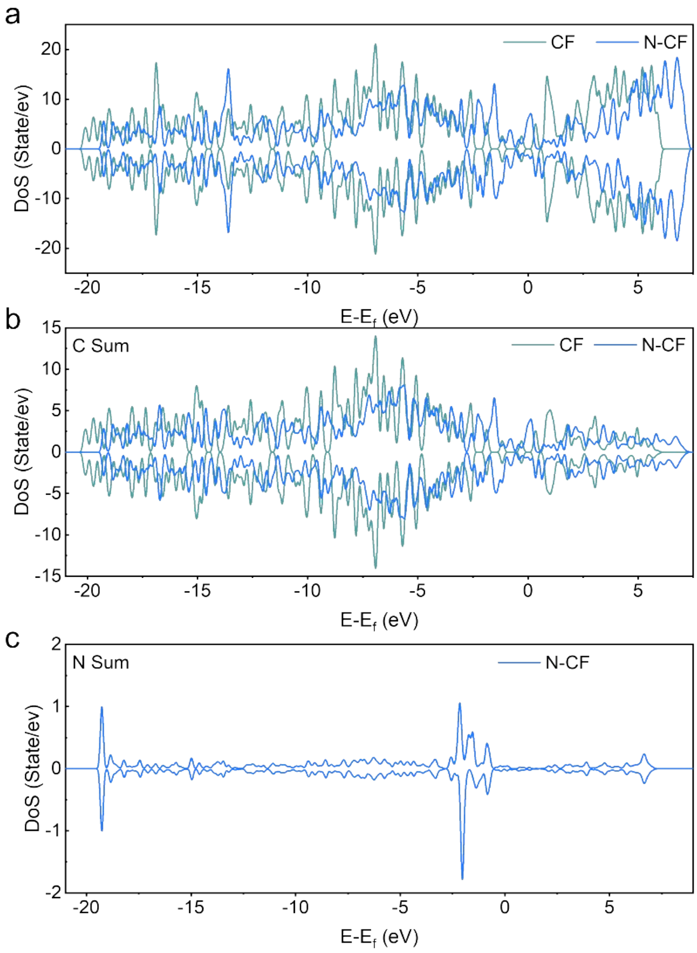

Figure S16 TDOS and PDOS plots calculated for the Potassium adsorption on Pyr-N and monolayer graphene models: (a) Total Density of States (TDOS) plot, (b) Carbon (C) Projected Density of States (PDOS) plot, and (c) Nitrogen (N) Projected Density of States (PDOS) plot.

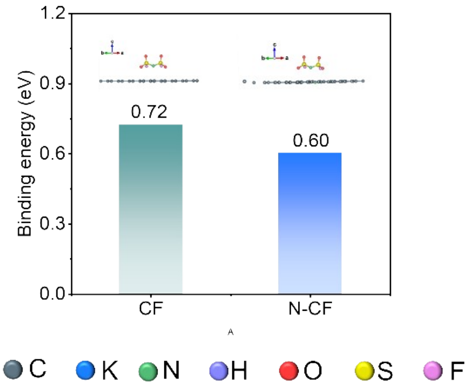

Figure S17 Bond energy changes ΔE_b based on DFT results of defluorination CF and N-CF.

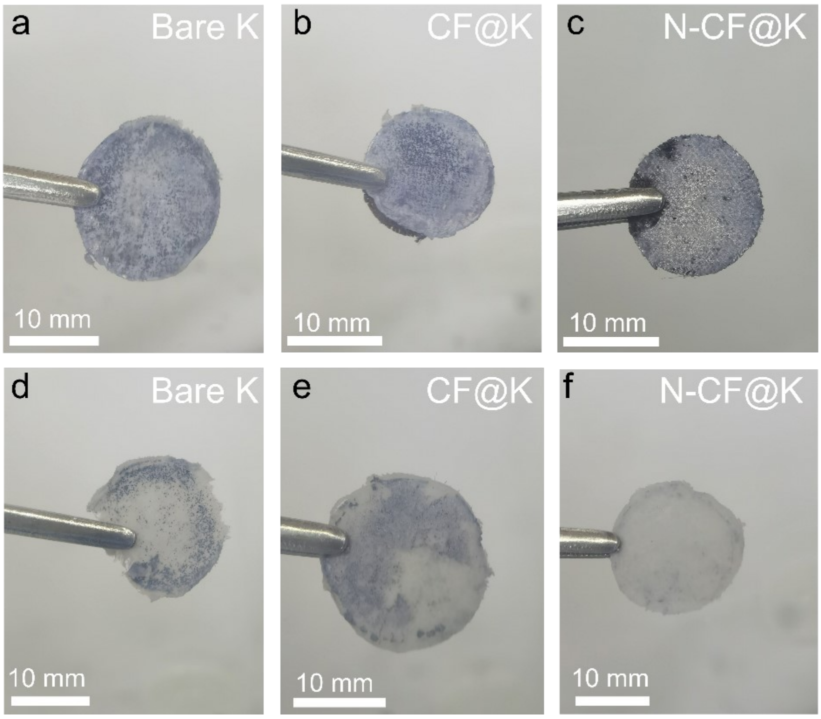

Figure S18 Front and side views of the electrodes and separators after 100 cycles at 0.5 mA cm^{-2}/0.5 mAh cm^{-2}: (a,d) Bare K, (b,e) CF@K, and (c,f) N-CF@K.

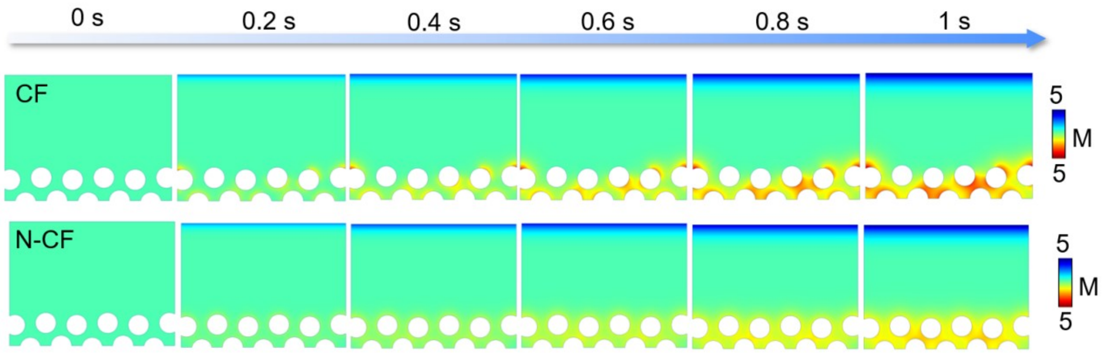

Figure S19 FEA of CF and N-CF during K plating process.

Table S1 Cycling performance of all the host materials for K metal anode in symmetric cells.

Host	Combination method	Current capacity density (mA cm^{-2}/mAh cm^{-2})	Cycle life (h)	Rate Performance (mA cm^{2}/mAh cm^{-2})	Ref
N-CC	Adsorption (100 °C /2 s)	0.5/0.5<br>0.5/1	4300<br>2000	9/1	This work
CBC	Adsorption (100 °C /2 s)	0.5/0.5<br>1/1	1400<br>1200	5/0.5	[S1]
Co–CNFs	Adsorption (100 °C /1.5 s)	0.5/0.5<br>2/1	1300<br>500	2/1	[S2]
MSCNF	Adsorption (150 °C /1 s)	1/1	800	5/1	[S3]
Ag-CC	Electrodeposition	0.5/0.5	700	2/0.5	[S4]
CC	Adsorption (80 °C /4 s)	1/1	350	5/1	[S5]
GIC	facile mixing	0.4/0.4	700	0.4/0.4	[S6]
DN-MXene/CNT	Adsorption (80 °C /100 s)	0.5/0.5	>300	2/0.5	[S7]
rGO/3D-Cu	Adsorption (300 °C /6 s)	0.5/0.5	~200	2/0.5	[S8]
Sn/3D-K	Electrodeposition	0.2/1<br>1/1	~50<br>~50	1/1	[S9]
ACM	Adsorption (100 °C)	1/1	230	5/1	[S10]
Al@Al	Electrodeposition	0.5/0.5	440	3/0.5	[S11]
Al@G	Rolling	0.5/0.5	1000	2/0.5	[S12]
Cu3Pt–Cu	Punch	0.5/1	300	0.5/1	[S13]
Pd/Cu foam	Punch	1/1	130	1/10	[S14]
SA-Co/HC	Adsorption	0.5/0.5	2500	5/0.5	[S15]
Cu/SKS	Electrodeposition	1/1	900	2/2	[S16]
hoCNT	Electrodeposition	0.5/4	1000	2/2	[S17]

Bi_{80}/NrGO	Adsorption	0.2/0.1	3000	1/0.1	[S18]
OPCMs	Electrodeposition	1/1	800	4/4	[S19]
rGCA	Adsorption	0.5/0.5	400	2/0.1	[S20]
NiCo/NOGC	Electrodeposition	3/0.5	~190	7/0.5	[S21]
RGO	Fuse-mixing	0.5/0.5	500	2/0.5	[S22]
3D-K_3Bi	Fuse-mixing	0.5/1	450	0.5/1	[S23]
MoS_2/CC	Electrodeposition	0.5/1	700	4/0.5	[S24]
Au/Cu foam	Punch	1/1	350	0.5/10	[S25]
K_xP_y	Rolling	0.5/0.5	550	5/0.5	[S26]
Oxygen-carbon cloth	Adsorption (90 °C / 0.7 s)	0.5/0.5<br>1/0.5	5500<br>3500	3/0.5	[S27]

References

[S1] M. Zhou, W. Qi, Z. Hu, M. Cheng, X. Zhao, P. Xiong, H. Su, M. Li, J. Hu and Y. Xu, ACS Appl. Mater. Interfaces, 2021, 13, 17629-17638.

[S2] L. Wang, H. Wang, M. Cheng, Y. Hong, M. Li, H. Su, J. Sun, J. Wang and Y. Xu, ACS Appl. Energy Mater., 2021, 4, 6245-6252.

[S3] S. Li, H. Zhu, Y. Liu, Z. Han, L. Peng, S. Li, C. Yu, S. Cheng and J. Xie, Nat. Commun., 2022, 13, 4911.

[S4] J. Zhang, Y. Li, L. Zhu, X. Wang and J. Tu, Chem. Eng. J., 2022, 449,137659.

[S5] J. Meng, H. Zhu, Z. Xiao, X. Zhang, C. Niu, Y. Liu, G. Jiang, X. Wang, F. Qiao, X. Hong, F. Liu, Q. Pang and L. Mai, ACS Nano, 2022, 16, 7291-7300.

[S6] J. Zhang, Y. Li, L. Zhu, X. Wang and J. Tu, Energy Storage Mater., 2021, 41, 606-613.

[S7] X. Tang, D. Zhou, P. Li, X. Guo, B. Sun, H. Liu, K. Yan, Y. Gogotsi and G. Wang, Adv. Mater., 2020, 32, e1906739.

[S8] P. Liu, Y. Wang, Q. Gu, J. Nanda, J. Watt and D. Mitlin, Adv. Mater., 2020, 32, e1906735.

[S9] M. Ye, J. Y. Hwang and Y. K. Sun, ACS Nano, 2019, 13, 9306-9314.

[S10] L. Qin, Y. Lei, H. Wang, J. Dong, Y. Wu, D. Zhai, F. Kang, Y. Tao and Q. H. Yang, Adv. Energy Mater., 2019, 9(29), 1901427

[S11] P. Liu, Y. Wang, H. Hao, S. Basu, X. Feng, Y. Xu, J. A. Boscoboinik, J. Nanda, J. Watt and D. Mitlin, Adv. Mater., 2020, 32, e2002908.

[S12] Y. Zhao, B. Liu, Y. Yi, X. Lian, M. Wang, S. Li, X. Yang and J. Sun, 2022, 34, e2202902.

[S13] J. Wang, J. Yuan, C. Chen, L. Wang, Z. Zhai, Q. Fu, Y. Liu, L. Dong, W. Yan, A. Li and J. Zhang, Nano Energy, 2020, 75,104914.

[S14] J. Wang, W. Yan and J. Zhang, Nano Energy, 2022, 96,101731.

[S15] D. Zhang, X. Ma, L. Wu, J. Wen, F. Li, J. Zhou, A. M. Rao and B. Lu, Adv. Energy Mater., 2022, 13(2),2203277.

[S16] J. Xie, Y. Ji, L. Ma, Z. Wen, J. Pu, L. Wang, S. Ding, Z. Shen, Y. Liu, J. Li, W. Mai and G. Hong, ACS Nano, 2023, 17(2),1511-1521

[S17] Y. Liu, Y. Shi, C. Gao, Z. Shi, H. Ding, Y. Feng, Y. He, J. Sha, J. Zhou and B. Lu, Angew. Chem. Int. Ed., 2023, 62, e202300016.

[S18] Y. Feng, A. M. Rao, J. Zhou and B. Lu, Adv. Mater., 2023, e2300886.

[S19] S. Xie, W. Xie, Q. Zhang, X. Cheng, X. Ouyang and B. Lu, Adv. Funct. Mater., 2023, 33(36), 2302880.

[S20] J. Zhang, D. Cai, L. Zhu, X. Wang and J. Tu, Small, 2023, 19(34), 2301119.

[S21] Q. Shen, Y. He and J. Wang, J. Mater. Chem. A, 2023, 11, 9829-9839.

[S22] J. F. Wu, W. Zhou, Z. Wang, W. W. Wang, X. Lan, H. Yan, T. Shi, R. Hu, X. Cui, C. Xu, X. He, B. W. Mao, T. Zhang and J. Liu, Adv. Mater., 2023, 35, e2209833.

[S23] S. Ye, L. Wang, F. Liu, P. Shi, Y. Yu, eScience, 2021, 1(1), 75-82.

[S24] Y. Lei, M. Chen, Y. Li, W. Zhang, D. Zhao, Q. Zhu, *Mater. Today Phys.*, 2023, *35*, 101141.

[S25] H. Li., Y. Liu, J. Wang, W. Yan, J. Zhang, *Chem. Asian J.*, 2022, *17*(15), e202200430.

[S26] P. Shi, S. Zhang, G. Lu, L. Wang, Y. Jiang, F. Liu, Y. Yao, H. Yang, M. Ma, S. Ye, X. Tao, Y. Feng, X. Wu, X. Rui, Y. Yu, Adv. Energy Mater., 2021, 11(5), 2003381.
